# Social determinants impact both viral infections and brain development

**DOI:** 10.1038/s41390-025-04292-7

**Published:** 2025-07-25

**Authors:** Zoe Kang, Jill Lebov, Ana Paula Hamad, Youssef Kousa

**Affiliations:** 1 Center for Precision Medicine and Genomics Research, Children’s National Hospital, Washington, DC USA; 2 RTI International, Social, Scientific, and Environmental Systems, Research Triangle Park, Durham, NC USA; 3 Universidade de São Paulo Faculdade de Medicina de Ribeirão Preto, Neurosciences and Behavioural Sciences, Ribeirao Preto, São Paulo, Brazil; 4 Division of Neurology, Children’s National Hospital, Washington, DC USA

## Abstract

**Abstract:**

Brain development is a complex process that proceeds from the embryonic stage into young adulthood. During the first three years, the brain rapidly develops and lays the groundwork for downstream structures. Social determinants of health (SDOH), including the wider set of forces and systems that shape everyday life, can have detrimental effects on the structure and function of the developing brain. Differences in the distribution of resources and governance at the global, national, and local levels, can create health disparities in infectious disease proliferation within and between communities and countries. Social determinants of infectious disease and brain development have been thoroughly researched independently, yet research on the interactions between these outcomes is limited. Here, we review the potential that social, economic, and environmental factors can coalesce to mitigate or exacerbate the effects of virally induced brain injury by either buffering against or adding to neurological disability. We synthesize research concerning SDOH, brain development, and viral infection and the interconnectedness between these important global health issues. We find that the same SDOH that impact brain development can also increase the risk of viral infection during pregnancy and adverse sequelae in the fetus, including damage to the developing brain, which can contribute to lifelong effects that reinforce health inequities.

**Impact:**

- Synthesizes the relationship between prenatal social determinants of health and prenatal viral infections, social determinants and brain outcomes, and their interrelationships.
- This review highlights the need to conduct further research to understand the pathway of these factors and quantify the contributions of each factor to the neurodevelopmental trajectory of a child exposed to prenatal viral infections.
- Details the social and environmental conditions that increase vulnerability to prenatal brain injury and decrease resilience to neurological developmental interruptions.

## Introduction

**Fig. 1:**
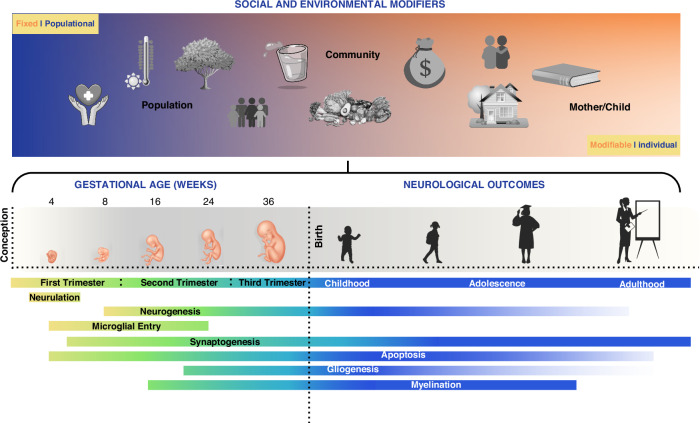
Exposure to populational, community, and individual social and environmental modifiers, collectively called social determinants of health, influence brain development and neurological outcomes. Throughout fetal development and the rest of an individual’s life, the neurobiology of the developing brain acts to form and maintain the structure of the brain. As these processes occur at a much higher volume during pregnancy and early childhood, early disruptions from social modifiers of health can be of particular concern.

The process of brain development begins within the first two to three weeks after conception and continues into young adulthood^1^. To successfully develop the 100 billion neurons that comprise the brain of a healthy newborn, the brain must grow at the rapid rate of 250,000 nerve cells per minute on average throughout pregnancy^2^. During this period of rapid division, neurogenesis, microglial entry, gyrification, synaptogenesis, and cell differentiation, among many other processes, the 3-mm neural tube becomes a human brain (Fig. 1)^2^.

The brain continues developing long after birth, with the prefrontal cortex fully developing in the mid-to-late twenties^1^. Though development continues into early adulthood, the developing brain is incredibly vulnerable in utero and during the first years of life^3^. During this time, critical foundations for language, social behavior, and emotion are formed^1^. Exposures during pregnancy and early life can permanently modify the brain structure and function through many mechanisms, including infection, inflammation, and epigenetic modifications^3^.

Congenital malformations of the central nervous system and associated developmental delays pose a significant burden to the child, the child’s family, and the broader health system. Among the myriad of causes of CNS abnormalities, vertically transmissible neuroinvasive viruses are particularly problematic because they are often difficult to detect or treat. When transmitted from mother to fetus, such viruses can cause congenital malformations, including fetal or neonatal brain development abnormalities^4,5^. Zika virus (ZIKV), an arbovirus spread by *Aedes (Ae.) Aegypti* and *Aedes Albopictus* mosquitos^6–9^, gained worldwide attention during the 2015-2016 epidemic, of which Brazil was the epicenter, due to the association between prenatal infection and an array of brain injuries and developmental abnormalities, the amalgamation of which has been termed Congenital Zika Syndrome (CZS)^10–12^.

CZS can include microcephaly, severe brain malformations, brain calcifications, hearing loss, vision problems, developmental disabilities, and facial disproportionality, among other congenital malformations and clinical features^4,5,10,13–19^. These brain injuries are often associated with developmental delays with variable severity but life-long impact on the infant and his/her family^17^. Approximately 6.22 billion people live in ZIKV-risk areas, which accounts for roughly 78% of the world’s population^20^. Research estimates there have been over 900,000 suspected cases reported since 2015, though due to often mild or no symptoms during infection, there have likely been many more cases^21^. To date, there are no approved vaccines or therapies to prevent or treat ZIKV infection or to mitigate brain injury and developmental disabilities associated with prenatal infection^22,23^. Whether the mother is symptomatic or asymptomatic, infants born to women infected with ZIKV during pregnancy have a 5–14% risk of developing CZS^4,24^.

It remains unknown why some children are born with no brain injury, minor brain injury, or CZS following prenatal ZIKV infection and what factors may influence subsequent developmental outcomes. This discrepancy may be partially attributable to Social Determinants of Health (SDOH), which the World Health Organization defines as the “non-medical factors that influence health outcomes, including the conditions in which people are born, grow, work, live, and age, and the wider set of forces and systems that shape the conditions of daily life^25^.”

**Fig. 2:**
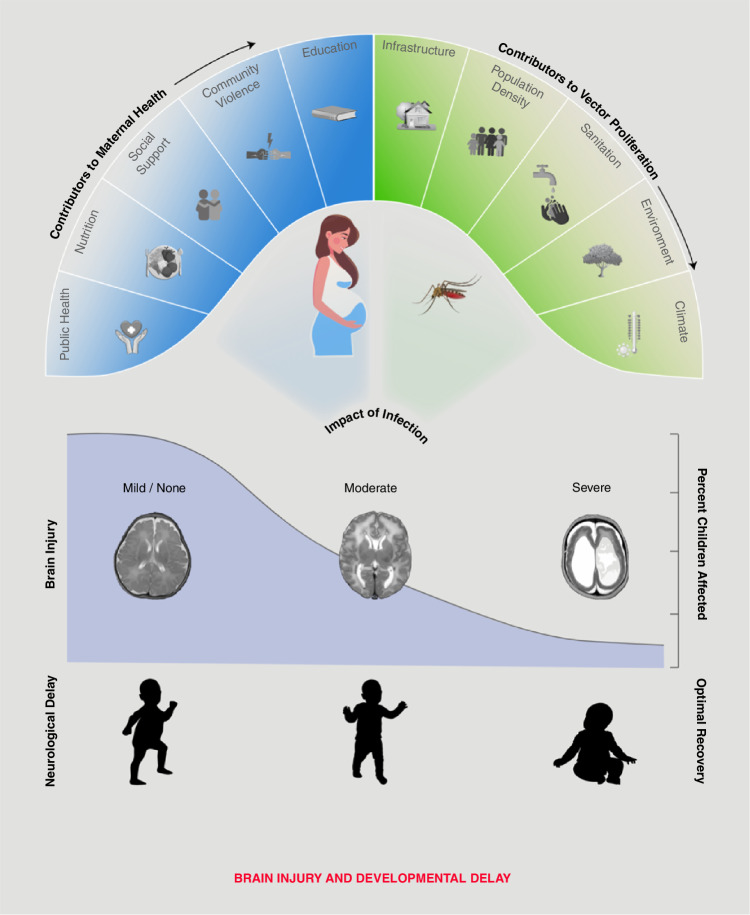
Social determinants of health influence vector density and proliferation. In turn, the number of vectors, human-vector contact, and number of pregnancies, all of which are impacted by other social determinants of health, influence the likelihood a woman will experience a prenatal Zika infection. These factors together typically inform the level of brain injury and developmental delays a child may have following prenatal Zika infection. The actual distribution of prenatal brain injury and neurological outcomes in the population is unknown. We used a sigmoidal curve to depict this distribution in line with current published literature on Zika virus and other viral infections, such as Cytomegalovirus.

Importantly, the distribution of brain injury and neurological outcome following prenatal viral infection remains largely unknown. As many as 80% of Zika virus infections are asymptomatic, contributing to large numbers of prenatal viral infections that go unidentified^26^. These undiagnosed cases of prenatal infection and lack of Zika virus testing in the absence of symptoms may lead to uncertainties regarding the number of cases of brain injury that are linked to prenatal viral infection^26^. Further, there is growing evidence that some brain injuries, especially mild and moderate cases, may have minimal outward symptoms^27,28^. Unless screened for in early life via imaging, mild and moderate cases of brain injury and altered neurological outcomes linked to prenatal viral infection that lack outward symptoms could go undiagnosed. We have depicted the distribution as a sigmoidal curve, in line with findings in the existing literature for other viral infections, like Cytomegalovirus (Fig. 2)^29–31^. However, the distribution may instead be bell-shaped, like many biological processes such as height and weight. The breadth of uncertain factors contributing to the available data on brain injury following prenatal infection makes the distribution of brain injury at the population level unknown and an area for future research.

SDOH includes the wider set of forces and systems that shape everyday life, creating avoidable health inequities within and between communities and countries^32^. The quality, quantity, and distribution of the socio-politico-economic conditions that make up SDOH play a large role in determining an individual’s health and well-being^33^. Though there is growing recognition of the importance of SDOH, underlying societal causes of poor health outcomes and health inequalities persist^33^.

Importantly, the effects of SDOH are deeply intertwined with one another, and there is very limited research illuminating their interactions or quantifying the contribution of each factor to the neurodevelopmental trajectory of a child. For example, a study by Ramphal et al. found that living in poverty can impact various areas of life critical to neurodevelopment, such as nutrition, physical activity, and maternal psychological state, resulting in decreased brain volume, developmental delays, behavioral issues, and neural network changes in fetuses^34^. Further, in a Brazilian cohort of 92 children aged 24–36 months, Neves et al. found that environmental and social factors showed a greater association with development than biological factors^35^. However, it is not known which factors are most influential and to what degree. SDOH has been thoroughly researched, but there is not yet an understanding of the relationship between SDOH, prenatal viral infection, and related brain injury and developmental outcomes.

Though it is beyond the scope of this review, it is notable that there is ample research on the biological mechanisms underpinning social and environmental exposures and their influence on health and brain development. Many studies have thoroughly examined the epigenetic relationship between SDOH and the biological mechanisms underlying social and environmental health discrepancies, including telomere length and rate of telomere loss, methylation, allostatic load, hormonal mediators, altered stress response, and altered protective microbiome^36–41^. The purpose of this review is to synthesize the relationship between SDOH and prenatal viral infections, SDOH and brain development, and the interrelationships between them. ZIKV and CZS in Brazil will be used as an example of these issues throughout the review.

## Introduction to social determinants of health

SDOH are deeply embedded in societies and have been shown to have a similarly substantial influence on health as genetic factors^42–44^. Though the underlying mechanisms are not yet fully understood, a dose-response relationship between health and some socio-demographic factors, such as income and education, has been observed^45^. SDOH acts in a cyclic and inheritable manner. A mother’s experiences in her childhood may be key determinants of her newborn’s outcomes, which in turn predict a newborn’s health outcomes in adulthood and so on (Fig. 1)^45–47^.

For this review, we focus on a range of social determinants at multiple levels, though the list of determinants is not exhaustive. At the individual level, these include Socioeconomic status (SES), which includes educational attainment and income, residence and home environment, personal transportation, social support, and family dynamics. For a community, critical factors include neighborhood infrastructure, green space, sanitation, population density, and access to food, clean air, and clean water. Population-level factors include access to quality and culturally competent healthcare, environment, climate, and public health infrastructure. Importantly, such levels are often difficult to separate, and bidirectional effects exist at all levels. These conditions exist on a spectrum from enabling to constraining and everywhere in between.

Social determinants that act at the population level are structural determinants. This means they are often well established in society, and to change them would require a great deal of structural and systematic alterations. These include climate change, air and water quality, and exposure to pollutants. Social determinants at the community and individual levels have much more potential to be modified and could serve as beneficial areas for intervention. Such factors may include nutrition, neighborhood resources, and social support. Intervening on modifiable factors has the potential to significantly alter the health of individuals and entire communities.

The effects of SDOH on health are incompletely characterized. Still, they are believed to work in an additive or multiplicative manner to improve or worsen individual, family, and community health and status. SES is a driving factor behind many of the SDOH related to prenatal infection and brain injury. SES is a person or group’s relative position in society based on their access to resources, power, and prestige^48^. SES encompasses income, education, occupation, and subjective perceptions of social status and class. Individuals living in low SES communities experience a double burden of increased risks of prenatal and childhood brain development deficiencies and increased risk of neuroinvasive vector-borne disease^48^.

## Socioeconomic status and brain development

Research has linked SES to alterations in the structure and function of the brain^49^. Lower SES can have adverse effects on brain development and developmental outcomes, including a reduction in brain volume and surface area, altered connectivity, and decreased cognitive function, behavior, and school performance^34,50–60^. Low SES is found to be associated with abnormal structural development of the brain in regions critical for the development of language, executive function, inhibitory control, and memory, thereby putting these individuals at increased risk for developmental delay^57,61,62^. Further, studies have found individuals from low SES families, when compared to their high SES counterparts, have significantly decreased average total volumes and reduced growth trajectories for grey matter, which is vital in cognition and behavioral regulation due to the presence of neural cell bodies, dendrites, and synapse that support the processing of information^62,63^.

Although SES exerts an independent effect on brain development, it can also serve as a proxy for exposure to environmental neurotoxicants and other chemicals that disrupt placental function and fetal and childhood development^64–66^. Low SES families are exposed to environmental toxicants, including organophosphates, heavy metals, chemical residues, and particulate matter at much higher concentrations and frequencies^59–63^ than their higher SES counterparts. It has long been known that the central nervous system is greatly affected by chronic environmental and toxic substance exposures during pregnancy, infancy, and childhood, which manifest as developmental abnormalities in brain structure, cognitive function, and behavior^64,65,67,68^. Individuals exposed to these environmental toxicants have been found to have reduced brain volume, altered motor and cognitive development, and neuroinflammation, including cell damage, cell death, and changes in synaptic plasticity^69–73^. Similar to SES alone, many studies have reported alterations in brain regions critical to executive function, attention, and emotion regulation following chronic early-life toxicant exposure^74,75^. The lasting effects of these abnormalities in the structure and function of the brain are often observed as severe behavioral problems, neurobehavioral disorders, and a lack of emotional control^76,77^.

By contrast, high SES is, unsurprisingly, associated with improved brain development. Evidence suggests advantaged SES can mitigate the effects of prenatal risk factors on developmental delays and aid in managing brain injury and delays through the availability of resources, including quality education, books, and specialized attention^50,57,78^. Families with greater financial resources can make investments in the development of their child by fostering cognitively stimulating environments and learning experiences, while disadvantaged families may only be able to invest in the child’s immediate needs^50,79–81^. Critical examination of the mechanisms through which high SES protects against worsening developmental delay among children born with CNS abnormalities could illuminate effective intervention strategies.

## Socioeconomic status and infectious disease

The incidence of viral infection and related sequelae is significantly increased among low SES communities due to increased population density and poor sanitation infrastructure^82,83^. *Ae. Aegypti*, the main vector of arboviruses like dengue, chikungunya, and Zika, reproduce in standing water, thrive in hot and humid climates, and have a short flight range of 150-200 yards^7,84–86^. Low SES communities that lack running water, sewage disposal, and proper infrastructure often have pools of standing water that become mosquito breeding sites^82,83,87–89^. Individuals who live in these urban or semi-urban communities spend much of their time in spaces that do not have protection from mosquitoes^90^. Communities with a confluence of these factors have the highest incidence of ZIKV infection (Fig. 2)^82,87^.

Limited by the short flight range, Zika vectors can only infect individuals close to their birth site and habitat. Therefore, neighborhoods with the highest population density offer the most opportunities for one vector to infect more people or increase person-to-person contact with infected individuals, thus experiencing the highest incidence of ZIKV infection^86^. Typically, the neighborhoods with the highest population density are occupied by individuals of low SES and lack necessary infrastructure^91^. In these communities, vector-borne viral infection can run rampant due to the surplus of vectors and the high density of individuals^87,91^.

Further, it has been observed that low SES women have higher fertility rates, thereby contributing to relatively higher rates of viral infection among pregnant women^44,82,92–96^. Importantly, previous literature has shown an increased incidence of Zika-induced brain injury, including CZS, following prenatal Zika infection among low SES mothers when compared to high SES mothers (Fig. 2)^7,43,82,97,98^. A recent study by Paixão et al. found evidence that socially vulnerable live births in Brazil, including individuals from regions with the most widespread poverty, the poorest quality of education, or lowest educational attainment, and the lowest SES, had the greatest risk of having a newborn with CZS^43^.

Souza et al. and Campos et al. found strong correlations between the distribution of Zika-related microcephaly and CZS with poverty and poor socioeconomic conditions, respectively^19,87^. Campos et al. further revealed that the Northeastern region of Brazil, the epicenter of microcephaly cases in the 2015–2016 epidemic, was also the region with the highest poverty index^87^. Notably, the well-documented co-occurrence of maternal malnutrition and protein deficiency in this region may provide potential mechanisms underlying these findings^99^. However, these findings are not confined to one region of the country. Another study conducted in multiple Brazilian states reported higher per capita gross domestic product is correlated with lower per capita rates of confirmed Zika-linked microcephaly cases^7^. Communities of low SES experience higher rates of Zika infection during pregnancy, increased risk of having a child with CZS, and other Zika-related CNS anomalies.

Importantly, as the climate changes, increased temperatures will allow breeding zones of *Ae. Aegypti* to increase in the geographic extent of transmission and transmission potential, putting more than half the world population in arbovirus-risk areas by 2030^100–102^. Climate models repeatedly predict climate change will exacerbate the global spread of vector-borne viral disease due to rising global temperatures and increased rainfall, fostering optimal climate conditions for vector proliferation and viral spread(Fig. 2)^12,82,103^. For example, models by Sadeghieh et al. comparing 2070–2100 to 2016 predict peak incidence of ZIKV to be over double that of 2016 and outbreak duration to be 50 weeks instead of 41^104^. Low SES communities that already experience the highest incidence of prenatal Zika infection and CZS will unequally bear this increased burden of viral disease.

Notably, though environment, climate, and climate change act at the population level, individual or community responses and ability to exist within the environment may vary due to a variety of factors, for example, income and employment. Climate change and the associated increased vector proliferation, spread of vector-borne viral disease, and virally induced prenatal brain injury are likely to worsen SES-linked viral infection disparities. Individuals or communities with access to more resources, better infrastructure, and sanitation may have decreased risk of arbovirus infection even while living in the same climate environment as their neighbors and neighboring communities (Fig. 2). A complete understanding of the role of SDOH in modifying outcomes has the potential to allow early intervention and preventative measures in efforts to reduce the health inequities experienced with CZS and other virally-induced syndromes.

## Sdoh as a modifier of developmental trajectories

We have described how individuals with low SES experience barriers to healthy brain development and an increased risk of prenatal viral infection, leading to increased incidence of brain injury and developmental delays or disabilities. Social-environmental factors, including neighborhood violence, social support, and access to healthy food, may also hinder their ability to overcome injury or disability or can exacerbate preexisting developmental deficits in childhood caused by low SES exposure during pregnancy or stressful gestation.

Neighborhood crime and violence, which are elevated in low SES communities, can mediate the effect of SES on pregnancy outcomes and alter the volume, connectivity, and structure of the neonatal brain and brain systems related to emotion processing, executive control, and reward pathways^105–108^. For example, Brady et al. reported a strong correlation between residing in a neighborhood with high violent and property crimes and weaker neonatal connectivity between the amygdala, hippocampus, and the thalamus-anterior default mode network^107^. Further, after controlling for family aggression exposure, community violence continued to predict reduced hippocampal volumes^105^. Living in a neighborhood with violence can modify the trajectory of childhood development even if the brain is developing otherwise normally^105,109^. Early exposure to neighborhood violence and crime affects the neonatal brain and cognitive development in children, including difficulties with memory, behavior, and emotion processing^110,111^.

Social support, the support accessible to an individual through connection to other people, groups, and the larger community, which includes networks of family, friends, neighbors, and community members, may vary by SES and urbanicity^112,113^. While this support can come from family, friends, and neighbors, it may also come from public health systems and government-supported social and economic aid programs.

Unstable social support systems have repeatedly been found to have both immediate and long-term adverse effects on behavioral, socio-emotional, and cognitive development^114–116^. Lack of social support or the presence of harmful or unsupportive home environments, including institutional rearing or foster care, is found to alter the structure and composition of the developing brain by reducing grey matter volume in areas implicated with reinforcement-based decision-making, emotion regulation, and autobiographical memory, including the prefrontal cortex, hippocampus, and cerebellum^113,117–121^. Grey matter disturbances in these regions may represent a long-term neurobiological risk factor for later psychopathology and heightened risk-taking^120^.

The effect of social support on the developing brain begins far before birth and may act as an enabling or constraining factor for neurodevelopment, beginning at contraception and continuing throughout childhood^122^. Maternal stress is associated with low SES due to the increased presence of stressors, including a lack of resources, crowded housing, and the presence of violence^123–125^. In the prenatal period, maternal stress has been linked to structural and functional abnormalities, including smaller brain volume, altered cortical thinning, and altered microstructure and functional connectivity in fetuses and infants^126–129^.

Conversely, a supportive social environment, thus reduced maternal stress, can mitigate the risks of poor social support on developmental delays by overcoming or lessening the effects of other SDOH^130^. For example, a study found that the presence of nurturing parents or a supportive social network can buffer against the adverse effects of growing up in neighborhoods with high violence and crime^131^. Though SDOH modifiers, when combined, frequently exacerbate health issues, even one positive modifier has the potential to mitigate some adverse health outcomes.

Food poverty is the inability to acquire or afford an adequate quantity or quality of food to meet one’s basic needs and is an existing problem in underserved communities worldwide^132,133^. Many developmental trajectories that begin and accelerate in pregnancy and early life depend on adequate nutrition^38,134–136^. Protein, minerals, and vitamins are critical for early fetal brain development processes, including the closing of the neural tube and myelination^135^. Malnutrition can cause the initial deficit critical for brain development, but it likely also hinders the ability of a child to overcome developmental delays of continuously exposed to poor nutrition. It has been associated with impaired early childhood cognitive deficits, developmental delays, poor school achievement, and behavioral problems^137–140^. Galler et al. described research showing that early childhood malnutrition has been associated with long-term developmental abnormalities, including increased schizotypal personality, lowered IQ, increased neuroticism, decreased extraversion, openness, agreeableness, and conscientiousness, and deficiencies in protein and certain micronutrients specifically lead to other poor cognitive and behavioral outcomes^136^.

Low SES is associated with limited access to healthy foods through limited money and residence in a food desert or food swamp^132,133^. From 2020 to 2022, the percentage of households in Brazil with children under ten affected by hunger doubled from 9.5% to 18.1%^141^. Though malnutrition is not unique to Brazil, the increase in hunger coupled with increased risk of arboviral diseases is likely to put children at a disadvantage from conception through adolescence, with barriers to fetal brain formation and difficulties overcoming existing brain injuries and delays.

## Conclusion

The effects of SDOH exist on a spectrum from enabling to constraining, and their effects influence and exacerbate one another. Namely, SES influences many other factors, including neighborhood violence and exposure to toxicants, neighborhood infrastructure, violence, population density, nutrition, and sanitation. The factors that contribute to the increased risk of brain injury or developmental deficit are the same risk factors for arboviral proliferation and act as barriers to overcoming an existing brain injury or developmental delay. These factors tend to be inherited from one generation to the next, thereby deepening the roots of health inequities.

The same communities that lack resources to overcome brain injury and developmental delays are the ones faced with increased incidences of virally induced prenatal brain injury. This glaring health inequity provides reason to further investigate the potential interacting effects of social determinants on prenatal viral disease and brain development, and to develop feasible, sustainable solutions to mitigate these effects. Individuals or communities with access to more resources and better infrastructure, or who minimize their exposure to the elements, for example, by working indoors or installing window screens, may have decreased risk of arbovirus infection even though they live in the same environment and climate as others. Importantly, the presence of even one enabling modifier has the potential to mitigate against the effects of other constraining ones.

Studies are often limited by an inability to isolate these factors or tease variables apart to understand the source of health discrepancies, if one exists. Although the investigation of multiple social-environmental factors is a challenge in research, new methods have been developed for evaluating complex systems of social, economic, and environmental factors on the health of individuals and communities^142–144^. Ignoring the context in which an individual can access health prevention and improvement hinders the development of future interventions. More research is needed to understand the entirety of the pathways through which these factors interact and to be able to quantify the contribution of each factor alone and in combination to the neurodevelopmental trajectory of a child exposed to prenatal viral infection. A complete understanding of such modifiers has the potential to inform early intervention and preventative measures in efforts to reduce the health inequities experienced with CZS or severe brain injury.
